# Obesity, starch digestion and amylase: association between copy number variants at human salivary (*AMY1*) and pancreatic (*AMY2*) amylase genes

**DOI:** 10.1093/hmg/ddv098

**Published:** 2015-03-18

**Authors:** Danielle Carpenter, Sugandha Dhar, Laura M. Mitchell, Beiyuan Fu, Jess Tyson, Nzar A.A. Shwan, Fengtang Yang, Mark G. Thomas, John A.L. Armour

**Affiliations:** 1 School of Life Sciences, Queen's Medical Centre, University of Nottingham,Nottingham NG7 2UH, UK,; 2 Wellcome Trust Sanger Institute, Wellcome Trust Genome Campus, Hinxton, Cambridge CB10 1SA, UK and; 3 Research Department of Genetics, Evolution and Environment, University College London, Gower Street, London WC1E 6BT, UK

## Abstract

The human salivary amylase genes display extensive copy number variation (CNV), and recent work has implicated this variation in adaptation to starch-rich diets, and in association with body mass index. In this work, we use paralogue ratio tests, microsatellite analysis, read depth and fibre-FISH to demonstrate that human amylase CNV is not a smooth continuum, but is instead partitioned into distinct haplotype classes. There is a fundamental structural distinction between haplotypes containing odd or even numbers of *AMY1* gene units, in turn coupled to CNV in pancreatic amylase genes *AMY2A* and *AMY2B*. Most haplotypes have one copy each of *AMY2A* and *AMY2B* and contain an odd number of copies of *AMY1*; consequently, most individuals have an even total number of *AMY1*. In contrast, haplotypes carrying an even number of *AMY1* genes have rearrangements leading to CNVs of *AMY2A*/*AMY2B*. Read-depth and experimental data show that different populations harbour different proportions of these basic haplotype classes. In Europeans, the copy numbers of *AMY1* and *AMY2A* are correlated, so that phenotypic associations caused by variation in pancreatic amylase copy number could be detected indirectly as weak association with *AMY1* copy number. We show that the quantitative polymerase chain reaction (qPCR) assay previously applied to the high-throughput measurement of *AMY1* copy number is less accurate than the measures we use and that qPCR data in other studies have been further compromised by systematic miscalibration. Our results uncover new patterns in human amylase variation and imply a potential role for *AMY2* CNV in functional associations.

## Introduction

The human amylase gene cluster is a highly repetitive, copy number-variable region that has undergone expansion in gene number specifically in the human lineage (1) and has been highlighted in genome-wide studies as an unusually extensive and important copy number variation (CNV) (2,3).

**Figure 1.**
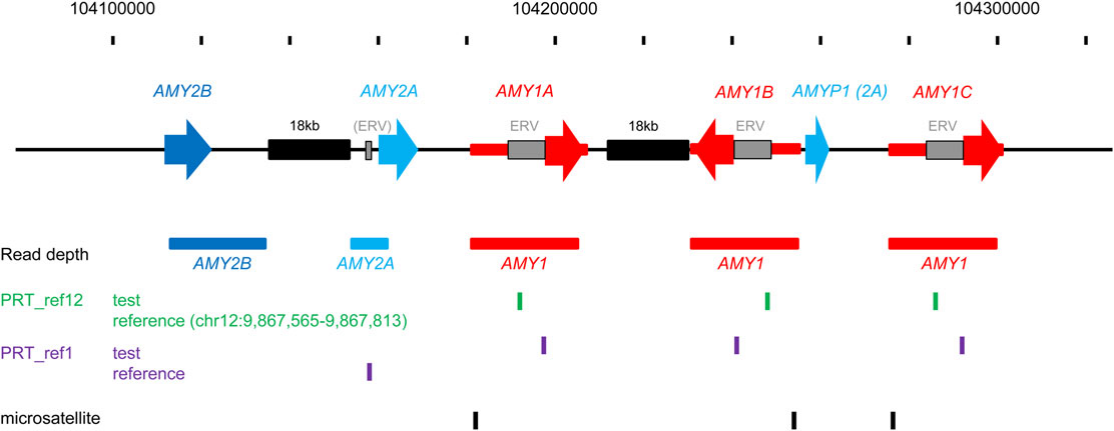
General overview of the human amylase gene region, showing the positions of assays used. The corresponding region of the human reference (hg19) assembly shown includes three copies of *AMY1* (*AMY1*A/1B/1C) and one copy each of *AMY2A* and *AMY2B*. In each case, the broad arrow indicates the position and orientation of the gene, and the more extensive region of near-perfect sequence identity around each copy of *AMY1* is shown as a narrower red rectangle. Exons 4–10 of *AMY2A* are repeated in the truncated pseudogene *AMYP1* between *AMY1*B and *AMY1*C. The black ‘18 kb’ rectangles indicate two near-identical regions with multiple sequence similarity to other parts of the locus. The ‘read-depth’ annotation shows the regions used for read-depth analysis of *AMY2B* (dark blue), *AMY2A* (light blue) and *AMY1* (red), indicating coherent intervals not compromised by high-sequence identity to other locations. The ‘PRT_ref12’, ‘PRT_ref1’ and ‘microsatellite’ annotations indicate the predicted positions of PCR products used in our copy number measurements at this locus.

The human (hg19) reference assembly shows a structure including three *AMY1* gene repeats, corresponding to the positionally defined *AMY1*A, *AMY1*B and *AMY1*C copies in the original assembly of the locus sequence (4), in which the middle (*AMY1*B) copy is in inverted orientation (Fig. 1). In addition to a few small indels, these three copies of the *AMY1* gene are more than 99.9% identical in DNA sequence to one another and a similar sequence identity extends for a region of about 26.5 kb around each copy, implying that this corresponds to the underlying copy-variable repeat unit of *AMY1*. The human pancreatic amylase genes *AMY2A* and *AMY2B* are present at the telomeric end of the same cluster. The *AMY1* gene sequence is 93.2% identical to *AMY2A* and 93.6% identical to *AMY2B*; *AMY2A* and *2B* genes share 94% sequence identity with each other.

The characterization of the human salivary amylase (*AMY1*) genes as CNV goes back more than 30 years to pedigree analysis of protein electrophoresis and of restriction fragments detected by Southern blot hybridization (5,6). These early studies showed that *AMY1* copy number correlates with salivary amylase concentration in saliva and characterized common amylase haplotypes containing odd numbers (1, 3, 5, 7, etc.) of *AMY1* genes (4,7–9) differing by pairs of *AMY1* gene units. Variation consisting only of haplotypes with odd numbers of *AMY1* predicts that diploid individuals must have even numbers of *AMY1* units in total. More recent observations using array-comparative genomic hybridization (CGH) (2) or real-time polymerase chain reaction (PCR) methods (10,11) have confirmed the CNV and its association with enzyme expression, but have not demonstrated a predominance of genotypes with even numbers of *AMY1*. In some of these studies, fibre-FISH images have shown structures with as many as 12 *AMY1* repeats in both tandem and inverted orientation (2,11).

It is not clear why these views of *AMY1* variation differ, and, in particular, whether the failure of real-time PCR studies to detect a preponderance of even numbers reveals a limitation of their accuracy, or whether the predominantly odd-numbered haplotypes observed in early studies represented a misleading sample. Furthermore, although most attention has focussed on *AMY1* CNV, there have been reports of variation in copy number of the pancreatic *AMY2A*/*2B* genes (3,7,12,13); however, the high-sequence identity between *AMY1* and *AMY2* genes makes resolving these CNVs unusually difficult, and these *AMY2* variants, and their relationship to *AMY1* variation, have not been clearly characterized.

Previous work analysing amylase copy number has suggested that the variation is of functional importance. Array-CGH and real-time PCR methods were used by Perry *et al*. (11) to report an increase in *AMY1* gene copy number associated with starch-rich diets in humans, indicating adaptive change in this CNV in response to the adoption of agriculture. Independently, variable amplification of the (pancreatic) *AMY2B* gene has accompanied dog domestication, presumably as an adaptive response to increased dietary starch (14). Most recently, Falchi *et al*. (10) concluded from real-time PCR measurements that body mass index (BMI) was negatively associated with *AMY1* copy number, with low copy number predisposing to obesity.

In this work, we aimed to resolve outstanding questions about the nature of amylase gene variation in humans, by applying methods capable of resolving single repeat unit differences and methods that reliably distinguish *AMY1* sequences from the *AMY2* genes. We combine high-precision measurement methods with analysis of segregation to show that most amylase haplotypes worldwide contain odd numbers of *AMY1* repeat units, but that haplotypes carrying even numbers of *AMY1* are associated with rearrangements giving rise to CNV of the pancreatic amylase genes *AMY2A*/*2B*. As a consequence, we show that the copy numbers of *AMY1* and *AMY2A*/*2B* are numerically correlated. We support our conclusions with fibre-FISH analyses on combed DNA; comparison of our results with qPCR analyses of the same samples suggests that copy number measurements by qPCR may be insufficiently accurate and additionally subject to miscalibration in other published work.

## Results and Discussion

We aimed to characterize human amylase variation accurately by investigating the true range of variation at *AMY1*, in particular, to understand whether diversity on a wider scale could be understood in terms of the haplotype structure represented on the reference sequence assembly and demonstrated by restriction mapping (4); in parallel, we also sought to investigate the nature of copy number diversity at the pancreatic (*AMY2*) genes. Accurate characterization of *AMY1* CNV is technically demanding because of high copy numbers (up to 18) and the existence of highly similar paralogues (*AMY1*/*AMY2A*/*AMY2B*) that can introduce error into measurements relying on hybridization (such as array-CGH) or analysis of read-depth uncorrected for these sequence similarities. We therefore developed paralogue ratio tests (PRTs) (15) based on an endogenous retrovirus (ERV) sequence upstream of *AMY1* (16). The ‘PRT_ref12’ primers (17) simultaneously amplify products from the *AMY1* ERV and from a two-copy ERV locus on chromosome 12 to provide a high-resolution measure of *AMY1* copy number without interference from the *AMY2A*/*2B* sequences. The PRT_ref12 product is amplified from about 6 kb upstream of *AMY1*, and we found no clear examples of discrepancy between this and other measures of *AMY1* CNV. We also characterized a compound tetranucleotide microsatellite about 16.6 kb upstream of each copy of the *AMY1* gene, but still within the coherent block of sequence near-identity around *AMY1*; observed numbers and signal strengths of microsatellite peaks matched the copy number of *AMY1* genes from PRT and locus-specific analysis of low-coverage sequence reads from the 1000 Genomes Project (Supplementary Material, Figs S1 and S2). We were able to initially calibrate copy numbers using segregation in 16 three-generation CEPH pedigrees, for many of which only a single integer solution was consistent with the data. These calibrations were subsequently validated by read-depth data from the 1000 Genomes Project (Supplementary Material, Figs S1–S3) and by fibre-FISH (Supplementary Material, Fig. S8). Consequently, using combined analysis of microsatellite profiles and PRT data, we derived consistent estimates of *AMY1* copy number for 749 unrelated samples, made up of 269 from HapMap phases I and II, and 480 UK samples from ECACC HRC1-5 (Supplementary Material, Dataset S1).

**Figure 2.**
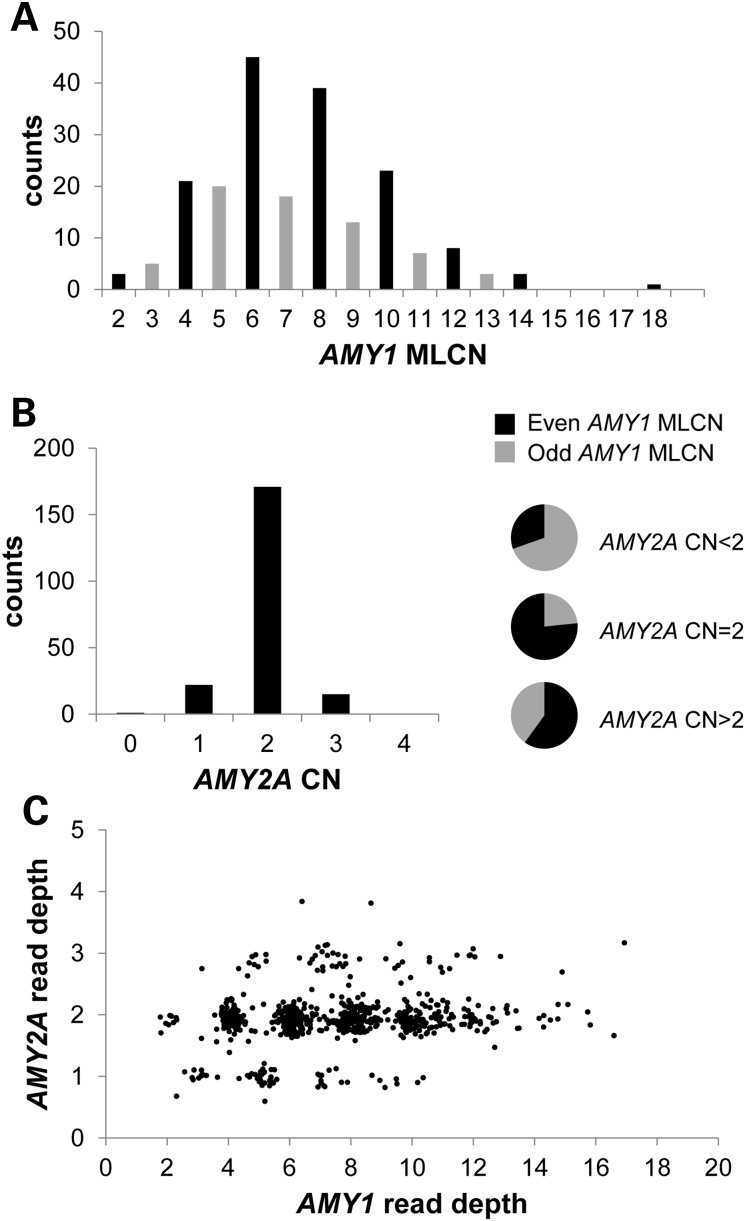
Most people have an even number of *AMY1*; odd numbers are associated with *AMY2A* CNV. (**A**) Integer *AMY1* maximum-likelihood copy number (MLCN) measured by PRT and microsatellite methods for 209 unrelated Eurasian individuals from the HapMap project. (**B**) *AMY2A* copy number (CN) for the same individuals, with pie charts illustrating the significant association between even numbers of *AMY1* and an *AMY2A* CN of 2 (*P* ≈ 5.3 × 10^−7^). (**C**) Estimates of read-depth-derived copy number estimates for *AMY2A* versus *AMY1* for 644 Eurasian samples from the 1000 Genomes Project; the association between (integer-rounded) even numbers of *AMY1* and an *AMY2A* copy number of 2 is significant (*P* ≈ 1.5 × 10^−24^).

For 207 of the HapMap samples, PRT and microsatellite copy number measurements for *AMY1* were supported by locus-specific analysis of low-coverage sequence reads from the 1000 Genomes Project (18–20) (Fig. 2 and Supplementary Material, Figs S3 and S4). Concordance with these read-depth estimates not only corroborated the accuracy of the PRT_ref12 and microsatellite-based measures for the samples we had typed (Supplementary Material, Fig. S3), but also validated the application of our read-depth measures to a wider range of samples from the 1000 Genomes Project.

**Figure 3.**
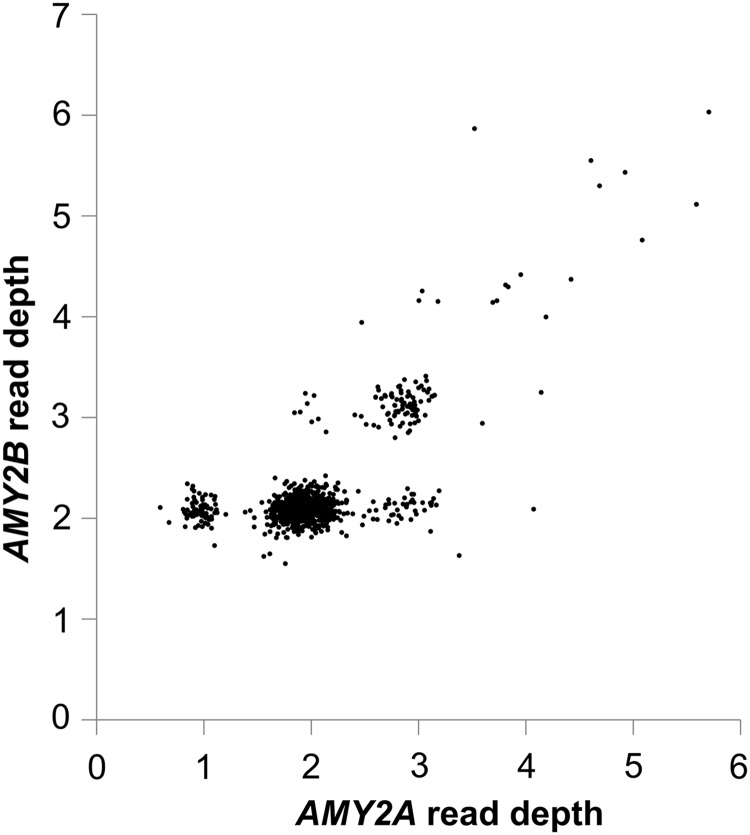
1000 Genomes read-depth demonstrates CNVs at *AMY2A* and *AMY2B*. Estimates of read-depth-derived copy number estimates for *AMY2B* versus *AMY2A* in 1047 samples from the 1000 Genomes Project, showing variant clusters corresponding to carriers of the *AMY2A* deletion (one copy of *AMY2A*, two copies of *AMY2B*), the *AMY2A/2B* duplication (three copies of *AMY2A*, three copies of *AMY2B*) and the *AMY2A*-only duplication (three copies of *AMY2A*, two copies of *AMY2B*). Eight samples have two copies of *AMY2A* and three copies of *AMY2B*, and this composition has been verified by segregation and other analyses in the case of NA11933.

**Figure 4.**
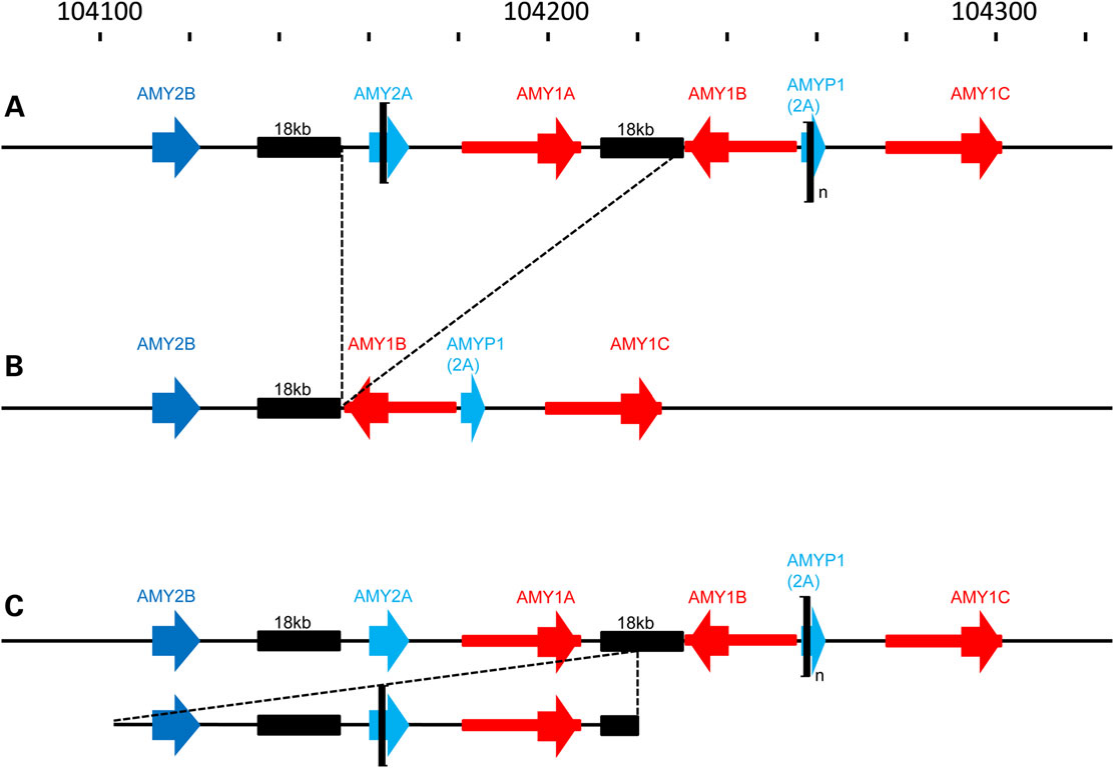
Inferred structures of *AMY1*/*AMY2* variants found commonly in European and Asian populations. The coordinates of the 3-copy haplotype in the hg19 reference assembly are shown at the top. Most haplotypes contain an odd number of *AMY1* and conform to the general structure shown in (**A**), containing one copy each of *AMY2A* and *AMY2B*, and variable numbers of a 95 kb variable-copy DNA unit containing two inverted copies of *AMY1* (bracketed) between exons 4–10 of *AMY2A* and the corresponding part of its pseudogene. The *AMY2A* deletion haplotype shown in (**B**) results from deletion of 75 kb containing both *AMY2A* and one copy of *AMY1*, thereby generating haplotypes carrying even numbers of *AMY1*, with a 2-copy example shown here. The haplotype in (**C**) shows the structure in which the *AMY2A* and *AMY2B* genes plus a copy of *AMY1* are included in a duplication unit of about 116 kb. The involvement of one copy of *AMY1* in the rearrangement again leads to haplotypes containing even numbers of the *AMY1* gene. Genes (and the pseudogene) are represented as arrows, with the extended region of sequence identity around *AMY1* shown as a narrow red rectangle. The black rectangles indicate two regions of about 18 kb between *AMY2A* and *AMY2B*, and between inverted copies of *AMY1*, that show high-sequence similarity. The position-specific nomenclature of *AMY1A/AMY1B/AMY1C* of Groot *et al.* (4) is adopted in this figure for clarity.

As a direct evaluation of the relationship between copy numbers of *AMY1* and *AMY2A*, we explored a second PRT assay (‘PRT_ref1’) that amplifies products from the *AMY1* ERV LTR (within 200 bp of the transcription start site) and uses the corresponding region in the ERV LTR upstream of *AMY2A* as a reference locus (17). We observed these measurements using PRT_ref1 usually matched estimates from PRT_ref12, but that some samples reproducibly gave substantially higher or lower apparent copy numbers of *AMY1* (Supplementary Material, Fig. S5). These observations suggested CNV at the *AMY2A* reference locus. We measured *AMY2A* and *AMY2B* copy number in the 207 HapMap samples for which *AMY1* copy number had been determined by both read depth and PRT. The data identified individuals with *AMY2A* and/or *AMY2B* variation, which was associated with an odd diploid total copy number of *AMY1*, in contrast to the majority of individuals with even copy numbers (Fig. 2). These observations ultimately led to the experimental and read-depth characterization of four distinct CNV classes affecting pancreatic (*AMY2*) genes (Figs 3 and 4): a deletion of about 75 kb affecting *AMY2A* (and *AMY1*), a duplication of about 116 kb including both *AMY2A* and *AMY2B* (and a copy of *AMY1*), higher-order expansions of *AMY2A* and *AMY2B* (found mostly in African individuals) and an independent duplication of *AMY2A* but not *AMY2B*. We estimate that the *AMY2A* deletion allele has a frequency of about 7% in Europeans, so that about 1 in 200 people will lack this gene product completely; in our data, sample NA12813 is homozygous for the *AMY2A* deletion. This sample was part of a collection of healthy volunteers for whom we have no detailed phenotypic information, and we are not aware of any phenotype associated with the *AMY2A* null phenotype in this individual or other deletion homozygotes. There have been reports of isolated pancreatic amylase deficiency (21,22), but the status of these individuals for the *AMY2A* CNV was not determined. In European samples, we found that the copy numbers of *AMY1* and *AMY2A* are significantly correlated (*r* = 0.24, *P* = 3.6 × 10^−6^; see Supplementary Material, Section 4). The different CNVs of *AMY2A* and *AMY2B* we have defined can also be discerned in analysis of read-depth data (Fig. 3), and the association between *AMY2A* CNV and odd total numbers of *AMY1* is especially clear in European read-depth data (Fig. 2C, Supplementary Material, Fig. S4 and Dataset S2).

The *AMY2* copy number-variant haplotypes most commonly found in European individuals are the duplication of *AMY2A* and *AMY2B* and the deletion of *AMY2A*. Our initial detailed characterization of the *AMY2A*/*2B* duplication began with the observation of a step change in read depth of duplication carriers in unique-sequence DNA upstream of *AMY2B*, defining the telomeric boundary of the CNV. We then discovered examples of sequences rearranged at this point by searching the NCBI Trace Archive, from which the other partner in the new junction sequence was deduced (see Supplementary Material for further details). A PCR junction-fragment assay (Supplementary Material, Fig. S10) showed that this rearrangement was specifically associated with duplication of *AMY2A* and *AMY2B*, demonstrating that the rearrangement involved these endpoints, separated by about 116 kb (Fig. 4C). This CNV appears to conform in its extent and general properties to CNVR266.13 of Conrad *et al*. (13) and to the copy number gain defined by Cooke Bailey *et al*. (12). Detailed read-depth and long-PCR characterization of *AMY2A* deletions, corresponding approximately to CNVR266.9 of Conrad *et al*. (13), showed that no new sequences or combinations are generated by the deletion, with the deletion corresponding approximately to removal of chr1:chr1:104 161 926–104 226 715 from the reference assembly (see Supplementary Material for details).

**Figure 5.**
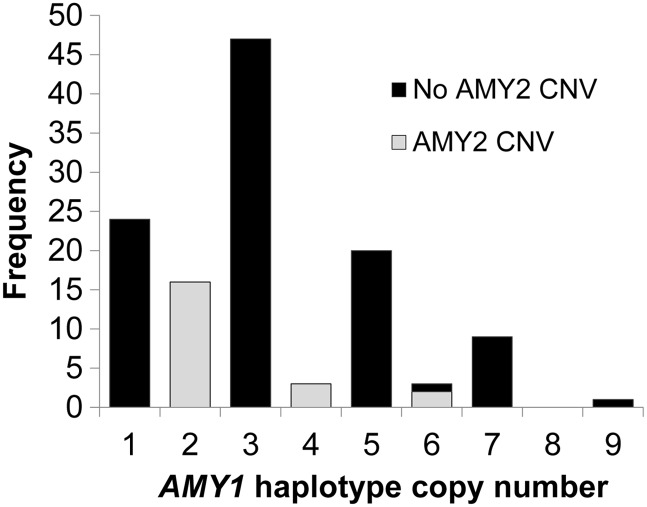
Haplotype copy numbers of *AMY1* inferred from pedigree segregation. Distribution of *AMY1* copy numbers in 123 haplotypes from 16 three-generation CEPH pedigrees. Colours distinguish haplotypes containing CNVs (2 or 0 copies) of *AMY2A* (all associated with even numbers of *AMY1*) from haplotypes containing one copy of *AMY2A* (the majority with an odd number of copies of *AMY1*).

Combining data from all populations, more than 60% of individuals had an even diploid number of *AMY1* genes and two copies each of *AMY2A* and *AMY2B*. However, a minority had an odd total copy number of *AMY1* in association with deletions or duplications of *AMY2* genes, and the frequencies of different *AMY2* variants differed markedly between population groups (Supplementary Material, Fig. S4). Among the regional population groupings used by the 1000 Genomes Project, East Asian populations display few *AMY2* variations and nearly all individuals have an even *AMY1* diploid copy number; deletions of *AMY2A* are common among the European and American samples, and duplications of *AMY2A*/*AMY2B* are at highest frequency in African samples. Segregation analysis in 16 European (CEPH) pedigrees allowed deduction of the microsatellite and *AMY* gene copy number content of haplotypes (Fig. 5, Supplementary Material, Fig. S6 and Dataset S3). About 80% of haplotypes contained odd numbers of *AMY1* copies, so that an individual with two such haplotypes had an even total number of *AMY1* genes. The content of these haplotypes conformed well to the haplotypes (H0, H1, H2) defined by Groot *et al*. (4,7,8,23). With specific assays for *AMY2A/2B* CNV (see Supplementary Material), we observed that the commonest of the minority haplotypes carry either a deletion of *AMY2A* (Fig. 4B) or a duplication of both *AMY2A* and *AMY2B* (Fig. 4C). One or other of these haplotypes is present in 20–25% of European individuals studied and both are strongly associated with even haplotype numbers of *AMY1* (Fig. 5).

**Figure 6.**
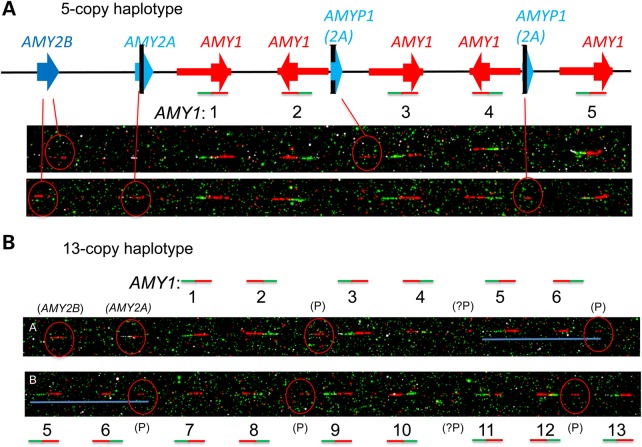
Fibre-FISH confirmation of 18 copies of *AMY1* in sample NA18972, with 13-copy and 5-copy haplotypes. The probes are identical to those used by Perry *et al.* (11) with the upstream (ERV) probe shown in green and the *AMY1* gene probe in red. Other sequences in the region which have >90% sequence identity to the *AMY1* gene probe hybridize more faintly and less reliably, interpreted here as *AMY2A*, *AMY2B* and the *AMY2A* pseudogene. (**A**) Two images of the 5-copy haplotype are interpreted using the diagrammatic representation shown in Figure 4. Note the faint and inconsistent cross-hybridization of the (red) *AMY1* gene probe to *AMY2B*, *AMY2A* and pseudogene sequences (circled). (**B**) For simplicity, the annotation of the 13-copy haplotype shows only sequentially numbered *AMY1* gene copies as paired red + green signals, with indication of the cross-hybridizing *AMY2B*, *AMY2A* and pseudogene (P) sequences circled in red. We estimate that the 13-copy haplotype, here presented as two overlapping images from the same molecule, spans about 670 kb between *AMY2B* and the last (13th) copy of *AMY1*. The overlap between images A and B is indicated by the horizontal blue line spanning *AMY1* repeats 5 and 6.

We used fibre-FISH to obtain further independent validation of haplotype structure and copy number for the highest *AMY1* copy number we observed, in Japanese sample NA18972. This sample had been assigned *N* = 14 by previous analyses, including fibre-FISH (3,11), and in other studies appeared to act as strong validation of qPCR measurements (11). Our measurement methods, including read depth, indicated a diploid *AMY1* copy number between 16 and 20 for NA18972, with *N* = 18 best supported overall (Supplementary Material, Fig. S2 and Datasets S1 and S2). Our fibre-FISH analysis strongly supported haplotype copy numbers of 13 and 5, giving a diploid total of 18 for this individual (Fig. 6). Given the predicted length of the 13-repeat haplotype allele (>500 kb) observed in this sample, we believe that the 10-repeat image shown by Perry *et al.* (11) is the result of a strand breakage during sample processing, as can be observed in some strands from this haplotype (Supplementary Material, Fig. S7). Supplementary Material, Figures S8 and S9 show other examples of fibre-FISH images supporting our inferred copy number and haplotype structure; multiple examples support a model for common haplotype structure containing odd numbers of *AMY1* differing by a pair of inverted *AMY1* copies, as predicted by simple extrapolation from the haplotypes containing 1, 3 or 5 copies of *AMY1* defined by Groot *et al*. (4) (see also Fig. 4A).

To confirm that our novel methods were an improvement on previous copy number measurement assays, we performed qPCR analysis on 269 unrelated HapMap samples using previously described methods (10) based on the assay of Perry *et al*. (11). Direct comparison of the raw copy number data showed that qPCR was less consistent than PRT; pairwise correlations between duplicate measurements for qPCR (*r*^2^ = 0.782) were lower than for PRT (*r*^2^ = 0.893). Furthermore, qPCR [normalized standard deviation (nSD) = 0.18] had higher measurement error than PRT (nSD = 0.13) and had a significantly different distribution of measured integer copy numbers for the same samples (*P* = 0.0176) (Supplementary Material, Dataset S1). The qPCR data did not identify a predominance of even copy numbers, suggesting that the qPCR assay was not sufficiently accurate to reflect the underlying genome structure of the amylase region. Together, these data suggest that the novel methods implemented here improve on existing amylase copy number measurement (see Supplementary Material, Dataset S1). Because of uncertainty about accuracy and calibration, we did not use qPCR in our overall evaluation of *AMY1* copy number.

In general, the appearance of data clusters corresponding to integer copy numbers is a strong indicator of CNV measurement accuracy and a useful basis for quality control of measurement data (24–27). Measurement of *AMY1* copy number by Perry *et al.* (11) generated qPCR data that failed to cluster around integer values or show any excess of even-numbered values. Our sample set included 46 samples from the HapMap Japanese (JPT) cohort measured by Perry *et al.*, and our read-depth and experimental methods generally report a higher *AMY1* gene copy number than Perry *et al.* (Supplementary Material, Fig. S2), suggesting that Perry *et al.*'s qPCR assay systematically underestimates *AMY1* copy number. If miscalibration in Perry *et al.* applies equally to all populations sampled, qPCR could still faithfully preserve a genuine difference in *relative* copy number estimates between high- and low-starch populations in spite of its inaccuracy. Individual assays for multiallelic CNVs can nevertheless be very sensitive to experimental artefacts differing systematically between sample sets, even with only minor variation in the quality and integrity of the DNA samples (24,28–30).

The difficulty of performing robust association studies with multiallelic CNVs is well illustrated by the case of the proposed associations between beta-defensin copy number and Crohn's disease. Two studies using qPCR measurements concluded that Crohn's disease is, in one study, significantly associated with low beta-defensin copy number (31), and in the second study with high copy number (32). In contrast, better-powered studies, in which greater attention was given to quality control of typing, found no evidence for association in either direction (25,33). Without objective validation of the accuracy of typing results, false-positive associations can arise from small but systematic biases in the outcome of typing assays between (for example) cases and controls, and it is clear that assays for multiallelic CNVs can be very sensitive to such bias (24,29).

The results of Perry *et al.* and, in particular, the designation of NA18972 as having 14 copies underpin qPCR calibration in the BMI association study of Falchi *et al.* (10). This miscalibration is sufficient to explain why Falchi *et al.* did not observe clusters around even-numbered integer values for *AMY1* copy number. However, the failure of their measurements to form any clusters strongly suggests that their qPCR assay does not reliably distinguish single repeat unit differences and that miscalibration is not the only source of error. Falchi *et al.* (10) report significant and reproducible associations between their measurements and BMI in different population samples, and even miscalibrated measurements could be sufficient to detect such an association, as long as the measurements are correlated with the true copy numbers. However, because of the correlation we observe between the copy numbers of *AMY1* and *AMY2A* genes, and Falchi *et al.*'s analysis of *AMY2A* copy number in only one of their cohorts, their data may still be consistent with the discovery of an association genuinely arising from *AMY2* CNV, for which *AMY1* copy number is acting as an indirect reporter. Different functional and physiological implications clearly arise depending on whether the observed association with BMI is primarily attributable to CNV in *AMY1* or *AMY2*. Similarly, although Perry *et al.*'s evidence for adaptation to starch-rich diets comes from analysis of *AMY1* copy number, differing frequencies of the *AMY2* variants described in this study would also be associated with corresponding shifts in *AMY1* copy number.

## Materials and Methods

### DNA samples

Ninety YRI samples, 45 JPT samples and 45 CHB samples from the International HapMap phase I, 180 CEPH European (CEU1/CEU2) samples from the International HapMap phase I and II (http://ccr.coriell.org, last accessed on 24 March 2015) and 480 random UK samples from the European Collection of Cell Cultures (ECACC) Human Random Control (HRC) panels 1–5 (https://www.phe-culturecollections.org.uk/collections/ecacc.aspx, last accessed on 24 March 2015) were used in the copy number measurement assays. The CEU samples used consist of 56 trios, 5 duos and 2 singletons. A further 116 individual CEPH family samples (http://ccr.coriell.org, last accessed on 24 March 2015) were used to infer segregation of the CEPH trios from the HapMap samples. The 16 CEPH families for which further samples were available to infer segregation were 1334, 1340, 1341, 1344, 1345, 1346, 1350, 1362, 1375, 1408, 1416, 1420, 1421, 1424, 1454 and 13292. All DNAs provided were extracted from lymphoblastoid cell lines.

### PRT_ref12 methods

PRT_ref12 primers amplify products from each copy of *AMY1* and from a reference locus at chr12: 9 867 565–9 867 813 (17). Reactions were performed with 1 µm each of primers HEX-ATCTAGTCCTTTTCTATCAATG and AGAAAATAAGTACATCTGTCAAG in a 20 µl reaction mixture consisting of 0.5 U Taq DNA polymerase (NEB) in a buffer with final concentrations of 50 mm Tris–HCl, pH 8.8, 12.5 mm ammonium sulphate, 1.4 mm magnesium chloride, 7.5 mm 2-mercaptoethanol, 125 μg/ml bovine serum albumin and 200 μm each deoxynucleotide triphosphate. PCR cycle conditions were: 23 cycles at 95°C for 30 s, 52°C for 30 s, at 70°C for 60 s followed by a final hold at 70°C for 30 min. PRT_ref12 gives products of 249 bp for the reference locus and 244 bp for the repeat region of *AMY1*. PCR products were mixed with 10 μl HiDi formamide with ROX-500 marker (Applied Biosystems, Warrington, UK). Fragment analysis was carried out by electrophoresis on an ABI3130 × l 36 cm capillary using POP-7 polymer with an injection time of 30 s at 1 kV. Copy number values were calculated by calibrating the ratios using HapMap CEU samples [NA11930 with *AMY1* copy number (CN) = 2; NA06993 with CN = 6; NA10852 with CN = 6; NA10835 with CN = 8; NA12248 with CN = 8; NA11931 with CN = 8; NA11993 with CN = 10; and NA07347 with CN = 11], which were included in every experiment in duplicate. These copy numbers gave reproducible and mutually consistent measurements with PRT, consistent microsatellite profiles and explained the observed segregation of copy number in third-generation (CEPH) pedigrees.

For four of these reference samples, 1000 Genomes data were available, and read-depth analysis gave copy number estimates of 2.06 for NA11930, 8.45 for NA11931, 10.17 for NA11993 and 11.88 for NA07347. A copy number of 10 for NA11993 was also confirmed using fibre-FISH (Supplementary Material, Fig. S8). Although both PRT and read-depth copy number estimates are subject to error, especially at relatively high copy number, the concordance of PRT_ref12 measurements with read-depth data (Supplementary Material, Fig. S3), and, in particular, the absence of widely discrepant copy number estimates provide reassurance that PRT_ref12 is not compromised by frequent polymorphism at the reference site on chromosome 12.

### Microsatellite typing

A TATC microsatellite within the amylase repeat unit was identified and the ratio between alleles was used to aid integer *AMY1* copy number assignment. PCR was performed with 1 µM each of primers FAM-ATTATCCTTTCACAGACAAAAG and TCCTCTAGGGTCATTCATTT to generate amplicons usually ranging from 249 to 277 bp in length, but in a few cases as long as 320 bp.

### Preparation of single DNA-molecule fibres by molecular combing and CNV validation by fibre-FISH

Four human lymphoblastoid B-cell lines (GM11993, GM12239, GM12813 and GM18972) were purchased from the Coriell Institute for Medical Research. Single-molecule DNA fibres were prepared by molecular combing (34) according the manufacturer's instructions (Genomic Vision). Briefly, the cells were embedded in a low-melt-point agarose plug (1 million cells per plug), followed by proteinase K digestion, washing in 1 × TE (10 mm Tris, 1 mm EDTA, pH 8.0) and beta-agarase digestion steps. The DNA fibres were mechanically stretched onto saline-coated coverslips using a Molecular Combing System (Genomic Vision).

Probe DNA was initially amplified from human genomic DNA using primer pairs CCTAGCCTGTTTTTGCAATTTTCTCT and TAAAATTTGGCTTTCCTTCCCTTGTA to amplify a 11158 bp product from the *AMY1* gene (11), TAAGCCTTGGGAAAGAAGTTGTCC and GCCCTTCCCAGCCTCTAGATAAAT to amplify an 8139 bp product from the *AMY1* ERV (11) and GTGAGCTAACCCCCTGTGTCAGA and CCTGAGTAGCATCATTGTAGTTCTCG to amplify a 6520 bp product from *AMY2A* (this work). To convert these products into FISH probes, purified long-range PCR products were first amplified using a GenomePlex^®^ Whole Genome Amplification (WGA) Kit (Sigma-Aldrich) following the manufacturer's protocols, then labelled using a modified WGA reamplification kit (Sigma-Aldrich) as described (35). For the fibre-FISH, approximately 500 ng of labelled DNA from each probe and 4 μg of human Cot-1 DNA (Invitrogen) were precipitated using ethanol, then resuspended in a mix (1:1) of hybridization buffer [containing 2× SSC, 10% sarkosyl, 2 m NaCl, 10% SDS and blocking aid (Invitrogen)] and deionized formamide (final concentration 50%). Coverslips coated with combed DNA fibres were dehydrated through a 70, 90 and 100% ethanol series and aged at 65°C for 30 s, followed by denaturation in an alkaline denature solution (0.5 m NaOH, 1.5 m NaCl) for 1–3 min, three washes with 1 × PBS (Invitrogen) and dehydration through a 70, 90 and 100% ethanol series. The probe mix was denatured at 65°C for 10 min before being applied onto the coverslips and the hybridization was carried out in a 37°C incubator overnight. The post-hybridization washes consisted of two rounds of washes in 50% formamide/2× SSC (v/v), followed by two additional washes in 2× SSC. All post-hybridization washes were done at 25°C, 5 min for each time. Digoxigenin-11-dUTP (Roche) labelled probes were detected using a 1:100 dilution of monoclonal mouse anti-dig antibody (Sigma-Aldrich) and a 1:100 of Texas Red-X-conjugated goat anti-mouse IgG (Molecular Probes/Invitrogen); DNP-11-dUTP (PerkinElmer) labelled probes were detected using with a 1:100 dilution of Alexa 488-conjugated rabbit anti-DNP IgG and 1:100 Alexa 488-conjugated donkey anti-rabbit IgG (Molecular Probes/Invitrogen); biotin-16-dUTP (Roche) labelled probes were detected with one layer 1:100 of Cy3-avidin (Sigma-Aldrich). After detection, slides were mounted with SlowFade Gold^®^ mounting solution containing 4′,6-diamidino-2-phenylindole (Molecular Probes/Invitrogen). Images were visualized on a Zeiss AxioImager D1 epifluorescent microscope. Digital image capture and processing were carried out using the SmartCapture software (Digital Scientific UK).

## Other Methods

Details of other methods and analyses used can be found in the Supplementary Material.

## Supplementary Material

Supplementary Material is available at *HMG* online.

## Funding

This work was supported by the Biotechnology and Biological Sciences Research Council (grant number BB/I006370/1 to J.A.L.A.), the University of Nottingham and the Wellcome Trust (grant number WT098051 to B.F./F.Y.). Funding to pay the Open Access publication charges for this article was provided by the BBSRC and the Wellcome Trust.

## Supplementary Material

Supplementary Data
