# Supplementary material for: Obesity, starch digestion and amylase: association between copy number variants at human salivary (AMY1) and pancreatic (AMY2) amylase genes
Source: Hum Mol Genet. 2015 Mar 18;24(12):3472–80. doi: 10.1093/hmg/ddv098 (PMC4498156; doi:10.1093/hmg/ddv098)
Supplement: Supplementary Data [file supp_24_12_3472__index.html]

Obesity, starch digestion and amylase: association between copy number variants at human salivary (AMY1) and pancreatic (AMY2) amylase genes — Obesity, starch digestion and amylase: association between copy number variants at human salivary (AMY1) and pancreatic (AMY2) amylase genes — Supplementary Data 

# Obesity, starch digestion and amylase: association between copy number variants at human salivary (*AMY1*) and pancreatic (*AMY2*) amylase genes

## Supplementary Data

Supplementary Data

**Files in this Data Supplement:**

- Supplementary Data - Pdf file
- Supplementary File 1 - xlsx file
- Supplementary File 2 - xlsx file
- Supplementary File 3 - xlsx file
