# Supplementary material for: Obesity, starch digestion and amylase: association between copy number variants at human salivary (AMY1) and pancreatic (AMY2) amylase genes
Source: Hum Mol Genet. 2015 Mar 18;24(12):3472–80. doi: 10.1093/hmg/ddv098 (PMC4498156; doi:10.1093/hmg/ddv098)
Supplement: Supplementary Data [file supp_ddv098_ddv098supp.pdf]

Supplementary Material for Carpenter *et al*., "Obesity, starch digestion and amylase: Association between copy number variants at human salivary (*AMY1*) and pancreatic (*AMY2*) amylase genes"

Contents

	Page
**Section 1: DNA samples and PCR methods**	**2**
**Section 2: Experimental determination of *AMY1* copy number**	**4**
**Section 3: Analysis of 1000 Genomes Project sequence data**	**6**
**Section 4: Properties of *AMY2* variants**	**8**
**Section 5: CEPH family segregation and haplotype analysis**	**11**
**Section 6: Comparisons with qPCR measurements**	**12**
**References (Supplementary Material)**	**14**
**Supplementary Figures S1-S10 and legends**	**15-24**

Section 1: Further details of PCR methods

PRT_ref1 methods

PRT_ref1 primers amplify a 122bp product from the ERV upstream of each copy of *AMY1* and a 141bp product from a reference site in the ERV in a corresponding position upstream of *AMY2A* (chr1:104159115-104159255). PCR was performed using 1µM each of For: HEX- CTTCTAAATCATAAGTTGATTT and Rev: CTTGTTTTATATTGTTTTCTATT, in a reaction mixture of 0.5U Taq DNA polymerase (NEB) in a buffer with final concentrations of 50mM Tris–HCl pH8.8, 12.5mM ammonium sulphate, 1.4mM magnesium chloride, 7.5mM 2-mercaptoethanol, 125µg/ml BSA and 200µM each dNTP. After an initial denaturation of 95°C for 5 minutes, 24 cycles of 95°C for 30s, 51°C for 30s and 70°C for 60s were performed, followed by a final extension step at 72°C for 20 minutes. Products were resolved by electrophoresis on an ABI3130xl 36 cm capillary using POP-7 polymer with an injection time of 30s at 1kV, and quantified using GeneMapper software (Applied Biosystems).

qPCR method for AMY1 copy number measurement

Copy number of *AMY1* was measured by quantitative PCR (qPCR) on 269 independent HapMap samples using a previously described method of duplex reactions of primers and Taqman probes for *AMY1* and *RNAseP* (Life Technologies) [1]. A 7900FAST Sequence Detector System (Applied Biosystems) was used to detect emitted fluorescence (FAM or VIC) from the probes during amplification. Values were extracted following analysis with the SDS software and diploid copy numbers were estimated by $\Delta\Delta$Ct method using the reference DNA sample NA18972 (Coriell Cell Repositories), which we observed to carry 18 copies of *AMY1* (see section 6), and NA18956 (Coriell Cell Repositories) which carries 6 copies of *AMY1.* All samples were run in triplicate to generate 3 raw estimated values for copy number in a single experiment. The average of these 3 values was used to estimate *AMY1* copy number for that sample. All 269 samples were measured by this qPCR procedure twice, allowing an assessment of reproducibility between duplicates. qPCR results are documented in detail in Dataset S1.

Junction fragment assay for AMY2A/2B duplication

The duplication assay is a three primer assay; one pair of primers directs amplification of a specific 323bp from the duplication junction (AMY2BR : TCAATTAGGAAATGAAGATATTGTTGA and AMY2BD:TGCCATAGACAAAATCTGTTGG). A third primer was included in the

assay to act as a control for successful amplification (AMY2BF: TCCTCCAAGACATTATTTTTGC) that in conjunction with AMY2BR would amplify a 424bp product from the region upstream of *AMY2B* in all subjects (see Supplementary Figure S10). Amplification from these primers was performed in a reaction mixture of 0.5U Taq DNA polymerase (NEB) in a buffer with final concentrations of 50mM Tris–HCl pH8.8, 12.5mM ammonium sulphate, 1.4mM magnesium chloride, 7.5mM 2-mercaptoethanol, 125µg/ml BSA and 200µM each dNTP. PCR cycle conditions were 36 cycles of 95°C for 30s, 48°C for 30s and 65°C for 1min. PCR products were visualized on a 2% (w/v) agarose gel.

Measurement of ratio between AMY2A and AMY2B

The ratio of *AMY2A* copy number to *AMY2B* copy number was measured using 1µM each of primers For: FAM-GATTTTTAATCAATACACATTTGC and Rev: ATAGTGACTTCCTTGCATTGGG in a reaction mixture of 0.5U Taq DNA polymerase (NEB) in a buffer with final concentrations of 50mM Tris–HCl pH8.8, 12.5mM ammonium sulphate, 1.4mM magnesium chloride, 7.5mM 2-mercaptoethanol, 125µg/ml BSA and 200µM each dNTP. PCR cycle conditions were 29 cycles of 95°C for 30s, 58°C for 10s and 61°C for 30s to generate amplicons of 163bp for *AMY2A* and 167bp for *AMY2B*. PCR products were mixed with 10µl HiDi formamide with ROX-500 marker (Applied Biosystems, Warrington, UK). Fragment analysis was carried out by electrophoresis on an ABI3130xl 36 cm capillary using POP-7 polymer, injecting at 1kV for 30s. GeneMapper software (Applied Biosystems) was used to extract the peak areas and calculate the ratio.

Measurement of ratio between AMY2A and AMY2A pseudogene

The ratio of *AMY2A* copy number to *AMY2A* pseudogene copy number was measured using 1µM each of primers For: HEX- CTGTAGGATAAGGAATGAGACA and Rev: GTATCGACTGAAATTCCTTGA in a reaction mixture of 0.5U Taq DNA polymerase (NEB) in a buffer with final concentrations of 50mM Tris–HCl pH8.8, 12.5mM ammonium sulphate, 1.4mM magnesium chloride, 7.5mM 2-mercaptoethanol, 125µg/ml BSA and 200µM each dNTP. PCR cycle conditions were 24 cycles of 95°C for 30s, 54°C for 30s and 70°C for 30s to generate amplicons of 197bp for *AMY2A* and 232bp for the *AMY2A* pseudogene. PCR products were mixed with 10µl HiDi formamide with ROX-500 marker (Applied Biosystems, Warrington, UK). Fragment analysis was carried out by electrophoresis on an ABI3130xl 36 cm capillary using POP-7 polymer with an injection time of 30s at 1kV. GeneMapper software (Applied Biosystems) was used to extract the peak areas and calculate the ratio.

Section 2: Experimental determination of *AMY1* copy number

Consistency of microsatellite peak profiles with AMY1 copy number

Initial observations indicated that each *AMY1* copy was matched by a copy of the upstream tetranucleotide microsatellite, so that the ratios of peaks in microsatellite allele profiles could be used to deduce *AMY1* copy number. As with all such ratio methods, the observed profiles could support more than one likely copy number (for example, a profile with three peaks in the ratio 2:1:1 would be consistent with a copy number of 4, but also with any other multiple of 4). The consistency of the microsatellite ratios with *AMY1* copy number was validated by analysing the peak ratios in samples with well-established copy number. Illustrative examples of microsatellite profiles for different *AMY1* copy numbers, alongside read-depth for the samples from 1000 Genomes Project sequence data, are shown in Figs. S1 and S2.

We were able to use the microsatellite profiles to deduce segregation in 16 CEPH pedigrees, which allowed us to define haplotypes of *AMY1* copy numbers unambiguously. Summary information on *AMY1* and *AMY2A* haplotype content in these Europeans is shown in Figure 5, and documented in detail in Dataset S3. In larger-scale testing we compared the observed microsatellite peak ratios with the ratios expected from the integer *AMY1* copy number derived from integrated analysis of microsatellite and other data (see below). We computed a normalized value for each observed microsatellite peak ratio by dividing it by the closest-fitting value predicted by the integer *AMY1* copy number. The distribution of 1488 normalized microsatellite ratios from 834 copy number evaluations had a mean of 0.974 and standard deviation of 0.097, suggesting that the microsatellite alleles produce PCR products in ratios that match *AMY1* in copy number closely. Among 749 different samples tested, we observed no instances of microsatellite profiles incompatible with reproducible evidence from PRT or other assays. We conclude that the microsatellite is a useful and accurate proxy of *AMY1* in the measurement of gene copy number.

Integration of data from PRT and microsatellite measurements

For each sample, PRT_ref12 ratios (up to four replicates) were combined with microsatellite peak areas to evaluate the most likely individual integer gene copy number by a maximum-likelihood approach, as previously described [2-4]. Comparison with read-depth and other data indicated that PRT measurements were well modelled by a Gaussian distribution with a mean corresponding to the true copy number and a standard deviation of about 18% of that mean value. Examination of microsatellite ratios from individuals with well-established copy number, again including read-depth data, shows that observed values matched well to the ratios predicted from the best-fitting integer split (see "consistency of microsatellite peak

profiles", above). The error of measurement for these values was consistently reflected in a standard deviation of about 9% of the mean.

These parameters were used in a maximum-likelihood framework for which the combined PRT measurements and microsatellite profiles were tested for consistency with each possible integer copy number from 2 to 24. As a rough indication of confidence, a "minimum ratio" (MR) for each outcome was assigned as the ratio of the probability associated with the most likely copy number (MLCN) to the next most likely [3]. In our dataset the median MR value was 11.28, and the interquartile range 2.92-80.9. Most uncertainty was associated with higher-copy number samples: among the 65% of samples assigned a copy number below 8, the median MR value was 36.24.

qPCR data, analysis and calibration

Our integrated analysis of *AMY1* copy number using PRT and microsatellite data did not include qPCR, which we measured in order to make a comparative evaluation of the measurement methods. There is reasonable concordance between duplicate independently estimated copy numbers using qPCR (r^2=0.7817), but weaker than the correlation between independent duplicate PRT measures (r^2=0.8933). NA18972 has previously been assumed to carry 14 copies of *AMY1,* and other analyses use this sample as a calibrator at N=14 [1]. Through our analysis (see section 6) we are confident that the true *AMY1* copy number of NA18972 is 18 and therefore the qPCR data we have shown in Dataset S1 is calibrated as such. We also tested calibration of our data using N=14 for this sample, which generated a different distribution of copy numbers and not simply a shift in the distribution, presumably because the calibration also uses a (correctly assigned) sample with N=6 [1]. Furthermore, comparisons between qPCR measures with PRT and read depth using NA18972 as a calibrator at N=14 generated correlations that do not pass through the origin (see Dataset S1), but transect the axis at 2.875. By contrast, calibrating using a value of N=18 for NA18972 gives a regression line passing closer to the origin, suggesting that when calibrating qPCR using N=18, all three methods estimate comparable copy numbers for the same samples, whereas using N=14 generates systematically different copy numbers for many samples tested by qPCR compared with PRT and read depth.

Section 3: Analysis of 1000 Genomes Project sequence data

Low-coverage phase 1 sequence (Illumina) data were downloaded from the 1000 Genomes web server (ftp://ftp.1000genomes.ebi.ac.uk/vol1/ftp/data/). We used whole-genome mapped reads available in `.bam` format for 1047 of the 1092 samples[5]. These samples included European samples (EUR) (n=365) [GBR (n=82), FIN (n=89), IBS (n=14), CEU (n=99), TSI (n=96)]; East Asian samples (ASN) (n = 280) [CHB (n=90), JPT (n=95), CHS (n=95)]; American samples (AMR) (n=175) [PUR (n=55), CLM (n=58), MXL (n=62)]; and African samples (AFR) (n=228) [ASW (n=59), LWK (n=83), YRI (n=86)] .

Different specific approaches were developed to measure the read depth of *AMY1*, *AMY2A* and *AMY2B*, using local GC-matched sequence that was not variable in copy number as a reference. In all cases, read counts were extracted using samtools [6] with the command `samtools view –c`, defining the intervals with a corresponding `.bed` file.

On the Feb 2009 (h37/hg19) genome assembly, *AMY1* and the surrounding DNA is represented by three near-identical repeats. Consequently, nearly all reads mapping to these regions were associated with mapping quality scores close to zero, and we therefore measured read depth of *AMY1* without applying any mapping quality threshold. To assess read depth for *AMY1*, we identified a 20kb region including the *AMY1* gene plus surrounding near-identical DNA sequence represented at all three corresponding positions (chr1:104190000-104210000, 104227213-104247214 and 104284138-104304150) on the assembly. All reads across these intervals were summed to give the aggregate read depth across this interval, which had an average length of about 20kb. *AMY1* copy number was estimated after comparison with a local 20kb region matched for GC content, combining reads mapping to the non-contiguous copy-invariant intervals chr1:104059996-104070000 and chr1:104460001-104469995.

On the Feb 2009 (h37/hg19) genome assembly, only exons 1-3 of *AMY2A* are uniquely represented, with exons 4-10 reiterated at the pseudogene region. We therefore measured *AMY2A* read depth using reads mapping to the interval chr1:104153700-104161939, which includes exons 1-3 of *AMY2A* and a unique 6.3kb region upstream. We applied a quality threshold (`-q20`), which was also applied to reads from the 40001bp local copy-invariant comparator interval (chr1:104045000-104085000). If a mapping quality threshold was not applied, the density of reads apparently mapping to the *AMY2A* region showed a simple correlation with increasing *AMY1* copy number; this correlation was not supported by experimental and other observations. Applying a mapping quality threshold resolved the relative read densities into discrete clusters, with most samples having 1, 2 or 3

copies. We therefore believe that the application of a mapping quality threshold is necessary for an accurate measure of true copy number for *AMY2A*.

For *AMY2B* we recorded reads mapping to the interval chr1:104114335-104135000, including *AMY2B* and surrounding unique sequences. We compared the density of reads to a 296kb copy-invariant region, which in aggregate had a similar GC content to the *AMY2B* interval (chr1:104000000-104100000 and chr1:104304000-104500000). Overall, these methods allowed us to derive consistent and credible estimates of copy number based on read depth for *AMY1*, *AMY2A* and *AMY2B*.

Plots of read depth summarizing the outcome of these analyses in 1047 samples are shown in Fig. S4, and the full output, including raw read counts for the relevant intervals, is given as Dataset S2.

Section 4: Properties of *AMY2* variants

AMY2A deletion variant

A deletion variant of *AMY2A* was initially detected experimentally on the basis of comparisons between PRT_ref12 and PRT_ref1 methods which use different reference loci (see Fig. S5). This was subsequently validated using read-depth data and measurement of *AMY2A*:*AMY2B* ratios. Using results from read-depth analysis, possession of fewer than 2 copies of *AMY2A* is common among EUR (14.8%) and AMR (11.4%) samples, but less frequent among AFR (1.75%) and ASN (1.79%). This variant is weakly but significantly associated with SNP variants; among 365 EUR samples, for example, r^2 = 0.30 and D′ = 0.72 with rs17014913 ($P \approx 1.12 \times 10^{-22}$). These SNP associations are consistent in different populations, suggesting that the same *AMY2A* deletion is present in all populations.

Fine-scale read-depth analyses suggested that the rearrangement was consistent with removal of about 75kb relative to the structure shown in in the reference assembly (Figure 4b); the deletion affected the whole of the *AMY2A* gene, but created no distinctive step-changes in read-depth that could be used to identify a boundary for the deleted region. We therefore investigated whether rearrangements involving the “18kb” repeated regions (see Figure 1) could result in “seamless” deletion of *AMY2A*, in which no new sequence junctions are created. We used long PCR to investigate the characteristic sequence arrangements downstream of *AMY1A*, *AMY1B* and *AMY1C*. This analysis demonstrated that the deletion allele lacked the junction corresponding to the sequence downstream of *AMY1B* (Charles Ducker, JT and JALA, data not shown), suggesting that the deletion could be modelled by the approximately 75kb deletion from the reference assembly between chr1:104,161,926 and chr1:104,226,715. This creates a seamless deletion because the deletion breakpoint is replaced by sequence (spanning the region downstream of *AMY1* to the 18kb repeat region) that corresponds precisely in sequence to a junction found in the opposite orientation in non-deleted alleles, identical to the reference assembly sequence downstream of *AMY1A* at around chr1:104,211,000.

AMY2A/2B duplication and higher-order variants

On the basis of experimental comparisons between the two PRT systems we analysed (PRT_ref12 and PRT_ref1, Supplementary Figure S5) we were alerted to common duplication variants of the *AMY2A* region. We subsequently confirmed and defined the extent of the duplication using read-depth analysis, which showed parallel duplication of both *AMY2A* and *AMY2B*. Out of 365 Europeans (EUR) analysed using read depth, 12% had more than 2 copies of both *AMY2A* and *AMY2B*, in most cases 3 copies of each. This variant was also common in African

(AFR, about 8.6% carriers) and American (AMR, 10.9%) samples, but was less frequent in Asian (ASN) samples (only 1 carrier out of 279 samples analysed). We used more detailed analysis of read depth to demonstrate that the duplicated region began at about chr1:104,103,733. From this starting point we found that this region was involved in a new junction sequence represented among NCBI Trace Archive sequences, including ti|551170146, ti|549705010 and ti|550249109. This indicated that the duplicated region included about 116kb, with the other extremity at about chr1:104,219,537. These examples were used to design a specific PCR assay for the junction fragment, and we observed that amplification of the novel junction correlated absolutely with evidence for duplication of *AMY2A* and *AMY2B* (see Supplementary Figure S10 and Dataset S1).

No sequence junctions are destroyed by the duplication, so this assay only identifies individuals carrying at least one copy of the duplication allele; furthermore, this assay can neither distinguish homozygotes from heterozygotes, nor differentiate between carriers of the duplication allele and individuals potentially carrying a derived higher-order amplification allele (see below). Analysis of the surrounding SNP data identified that the *AMY2A/2B* duplication allele is associated with rs12075086T in all populations (Cochrane-Armitage P value < 10^{-137}), and more strongly associated in AMR and EUR ($r^2 = 0.7$, P ≈ 5 x 10^{-83}) with the nested haplotype defined by rs77729677G.

In the AFR samples there are individuals who appear to have 5 or 6 copies of both *AMY2A* and *AMY2B*, and are therefore obligate carriers of at least one haplotype containing more than 2 copies of these genes. In nearly all individuals with more than 3 copies of *AMY2A* and *AMY2B*, the copy numbers of the two genes are equal, suggesting that there are higher-order (up to 5-fold) amplifications of a unit carrying 1 copy each of *AMY2A* and *AMY2B*, which in combination with single-copy and duplication alleles give rise to observed *AMY2A/2B* copy numbers of up to 6 per individual. Such higher-order amplifications appear to be present at a cumulative frequency of about 3-4% among the AFR samples, but we found only a single example of an individual with *AMY2A/2B* copy number both greater than 4 in AMR, and none in other populations. Possession of more than 4 copies of *AMY2A* and *AMY2B* is, like the *AMY2A/2B* duplication allele, associated with rs12075086T in AFR ($r^2 = 0.136$, D′ = 1, P = 2.72 x 10^{-11}). Furthermore, analysis of HapMap YRI trios suggests that there can be up to 5 copies each of *AMY2A* and *AMY2B* on a haplotype, and all examples of higher-order amplifications are associated with the same junction fragment as is found in carriers of the simple *AMY2A/2B* duplication. We therefore believe that these higher-order *AMY2A/2B* amplification alleles prevalent in Africa are likely to be secondary derivatives of the more widespread *AMY2A/2B* duplication allele described above and illustrated in Figure 4c.

AMY2A-only duplication variant

Read-depth data highlighted the presence of an additional duplication variant of *AMY2A* that was not accompanied by duplication of *AMY2B*, leading to 3 copies of *AMY2A* but only 2 copies of *AMY2B* in heterozygous carriers. This variant appeared to be at high frequency in the African (AFR) samples studied (12.7% carriers), but at lower frequency in AMR (2.3% carriers), ASN (2.5%) and EUR (0.55%). In AFR and AMR, duplication of *AMY2A* unmatched by *AMY2B* duplication is weakly but significantly associated with rs139011323T ($r^2 = 0.15$, $D' = 0.4$, $P = 4.4 \times 10^{-30}$), but not in ASN or EUR, in which rs139011323 is not polymorphic.

Correlation between AMY1 and AMY2 variation

Given the association of *AMY2A/2B* duplication and *AMY2A* deletion with haplotypes containing an even number of copies of *AMY1*, we considered that there could be a correlation between *AMY1* and *AMY2A* copy number, especially in European populations, in which both the *AMY2A* deletion and the *AMY2A/2B* duplication are common. We therefore examined the integer copy numbers for *AMY1*, *AMY2A* and *AMY2B* deduced from read-depth in 1000 Genomes Project data. Simple correlation between *AMY1* CN and *AMY2A* CN in the global sample is weak ($r = 0.069$, $P = 0.025$), but stronger in the EUR subset ($r = 0.24$, $P = 3.6 \times 10^{-6}$). European carriers of the *AMY2A* deletion have a lower mean (5.55) and median (5) copy number for *AMY1*, and carriers of the *AMY2A/2B* duplication a higher mean (7.43) and median (7) *AMY1* copy number, than individuals with 2 copies each of *AMY2A* and *AMY2B* (mean *AMY1* CN 6.7, median 6). We conclude that in European population samples, if *AMY2A* copy number had not been typed, an association genuinely due to *AMY2A* or *AMY2B* copy number could in principle be detected as a weak association with the copy number of *AMY1*.

Section 5: CEPH family segregation and haplotype analysis

AMY1 haplotype analysis

We examined the segregation of microsatellite alleles and diploid *AMY1* copy number in three-generation CEPH pedigrees. The microsatellite alleles are highly variable and informative and in all 16 families we typed segregation could be inferred unambiguously (Fig. S6 and Dataset S3). The segregation allowed us to determine 123 *AMY1* haplotypes, among which we observed a predominance of $(AMY1)_{odd}$ haplotypes (101 out of 123, 82.1%) with 21 of the 22 $(AMY1)_{even}$ haplotypes carrying either the *AMY2A* deletion or the *AMY2A/2B* duplication. The composition of the $(AMY1)_{odd}$ haplotypes had a much greater range of the microsatellite alleles than the $(AMY1)_{even}$ haplotypes. All microsatellite alleles (249bp, 253bp, 257bp, 261bp, 265bp, 269bp, 273bp, 277bp and 320bp) were represented in the $(AMY1)_{odd}$ haplotypes, whereas for the $(AMY1)_{even}$ haplotypes only 3 alleles (261bp, 265bp, 269bp) were commonly represented, with 2 alleles (273bp, 277bp) represented once each.

Ratio of AMY2A and AMY2A pseudogene

We took advantage of a 35bp indel that differs between *AMY2A* and the *AMY2A* pseudogene to verify consistency of the proposed haplotype structures (see Section 1 for PCR details). Although we found that the variants do not always act as paralogue-specific variants, the total number of (*AMY2A* + pseudogene) predicts a limited range of possible ratios. For example, 4-copy individuals conforming to the standard common haplotypes are predicted to have 2 copies of *AMY2A* and 1 copy of the pseudogene, which would be consistent with variant ratios of 1:2, or 2:1. In practice, among 31 HapMap samples tested which were known to contain 4 copies of *AMY1* and 2 copies of *AMY2A*, 25 gave pseudogene:*AMY2A* ratios in the range 0.46-0.57, 5 gave ratios in the range 1.95-2.12, and one was uninformative (ratio = 0). Although it is of limited value in copy number determination on its own, this test provides a usefully accurate confirmatory test of the predictions of haplotype composition.

Section 6: Comparison with published qPCR measurements

We had access to only some of the samples typed by qPCR in the Perry *et al.* study[7], namely those that formed the Japanese cohort of the HapMap phase I study. We examined these using both experimental (microsatellite and PRT) and (1000 Genomes Project) read-depth methods. PRT and microsatellite data for 45 HapMap phase I Japanese (JPT) samples typed by Perry *et al.* were generated. Excluding NA18987, for which Perry *et al.* report the highly anomalous *AMY1* copy number estimate of 0.645, we found that PRT/microsatellite measurements gave the same integer *AMY1* copy number as reported by Perry *et al.* for only 3 out of 43 samples typed in both studies. Our measurements were higher than those of Perry for all 40 discrepant samples, by an average of 2 repeat units per sample.

For 39 JPT samples we were able to estimate *AMY1* copy number using low-coverage sequence data from the 1000 Genomes Project. Microsatellite and PRT data showed good agreement with read-depth data, with 25 out of 39 agreeing exactly on integer copy number, and 13 of the remaining 14 differing by a single repeat unit. There was no strong tendency for differences in one direction, and overall the integer values from PRT/microsatellite data and read depth differed by a mean absolute value of 0.38 repeats. By contrast, there was again a systematic tendency for Perry *et al*.'s qPCR to underestimate the *AMY1* copy number; only 5 samples agreed with the integer copy number suggested by read depth, with the read-depth integer being a mean of 2.16 (median 2) repeats higher. In no case was the qPCR value higher than the read depth estimate. The tendency of the qPCR method used by Perry *et al.* to underestimate the *AMY1* copy number given by other methods is most clearly demonstrated by microsatellite profiles (Fig. S2).

Sample NA18972

We believe that the consistent tendency of Perry *et al.*'s qPCR to underestimate *AMY1* copy number is likely to result from the adoption of a (2-copy) chimpanzee reference standard, without corroboration from higher copy number calibrating human samples. The tendency is most clearly illustrated by their assignment of N = 14 to NA18972. Despite the apparent concordance of fibre-FISH (Figure 3a in Perry *et al*.[7]) and qPCR measurements, we have made several observations that call this integer assignment into question.

A series of 13 independent PRT_ref12-based estimates of *AMY1* copy number produced values in the range 14.8-20.6, with a mean of 18.1 and a median of 18.4. NA18972 was included in every qPCR experiment and produced a range of copy-number estimates ranging from 12.899-24.347, with a mean of 17.86 and a median of 17.36. NA18972 was not included in phase 1 of the 1000 Genomes Project study,

but low-coverage sequence data were available from which read-depth analysis resulted in an *AMY1* copy number estimate of 18.6. Microsatellite profiles consistently gave a good match to an integer value of 18 for this sample, with peaks in the ratios 1:9:2:4:2. The profiles obtained are incompatible with an *AMY1* copy number lower than 15 (Fig. S2).

To test for consistency with different copy numbers if NA18972 had two standard haplotypes containing odd numbers of *AMY1*[8] we used measurement of the ratio between the *AMY2A* gene and its pseudogene (see Sections 1 and 5 above). For pairs of standard haplotypes summing to diploid totals of 14, 16 and 18 *AMY1* genes, the predicted ratios of the pseudogene to *AMY2A* are 3.0 (6:2), 3.5 (7:2) and 4.0 (8:2) respectively. Six independent measurements of this ratio in NA18972 yielded results in the range 3.76-4.06, with a mean value of 3.89, again favouring a copy number of 18, and difficult to reconcile with a copy number as low as 14.

Finally, the fibre-FISH images shown in Figure 3a of Perry *et al*.[7] appear to show haplotypes of N = 10 and N = 4, with a single example shown of each haplotype. Haplotypes containing an even number of *AMY1* genes are uncommon, especially in Asian samples. The interpretation of 10/4 is made even more unlikely by read depth and experimental observations indicating that NA18972 has 2 copies of both *AMY2A* and *AMY2B*. Furthermore, although the structures observed in both haplotypes are dominated by inverted repeat pairs of *AMY1* as expected, neither haplotype shown in their Figure 3a[7] has the expected orientation of genes at the ends of each array. By contrast, our own fibre-FISH results demonstrating a copy number of 18, consisting of 13-copy and 5-copy haplotypes, conform both to the predicted haplotype structure and odd-numbered *AMY1* content (Figure 6). Even using combed DNA we observed that there were examples of structures corresponding to incomplete fragments from the 13-copy haplotype (Fig. S7). We therefore suggest that the images shown in Perry *et al.*'s Figure 3a[7] result from strand breakage, as might frequently happen with alkali denaturation methods applied to structures in excess of 500kb, and that our Figures 6 and S7 are a more faithful representation of the true haplotype structures in this sample.

References

1. Falchi M, El-Sayed Moustafa JS, Takousis P, Pesce F, Bonnefond A, et al. (2014) Low copy number of the salivary amylase gene predisposes to obesity. Nature Genetics 46: 492-497.
2. Aldhous MC, Abu Bakar S, Prescott NJ, Palla R, Soo K, et al. (2010) Measurement methods and accuracy in copy number variation: failure to replicate associations of beta-defensin copy number with Crohn's disease. Human Molecular Genetics 19: 4930-4938.
3. Khan FF, Carpenter D, Mitchell L, Mansouri O, Black HA, et al. (2013) Accurate measurement of gene copy number for human alpha-defensin DEFA1A3. BMC Genomics 14: 719.
4. Walker S, Janyakhantikul S, Armour JAL (2009) Multiplex Paralogue Ratio Tests for accurate measurement of multiallelic CNVs. Genomics 93: 98-103.
5. The 1000 Genomes Project Consortium (2012) An integrated map of genetic variation from 1,092 human genomes. Nature 491: 56-65.
6. Li H, Handsaker B, Wysoker A, Fennell T, Ruan J, et al. (2009) The Sequence Alignment/Map format and SAMtools. Bioinformatics 25: 2078-2079.
7. Perry GH, Dominy NJ, Claw KG, Lee AS, Fiegler H, et al. (2007) Diet and the evolution of human amylase gene copy number variation. Nat Genet 39: 1256-1260.
8. Groot PC, Bleeker MJ, Pronk JC, Arwert F, Mager WH, et al. (1989) The human alpha-amylase multigene family consists of haplotypes with variable numbers of genes. Genomics 5: 29-42.

Supplementary Figures and legends

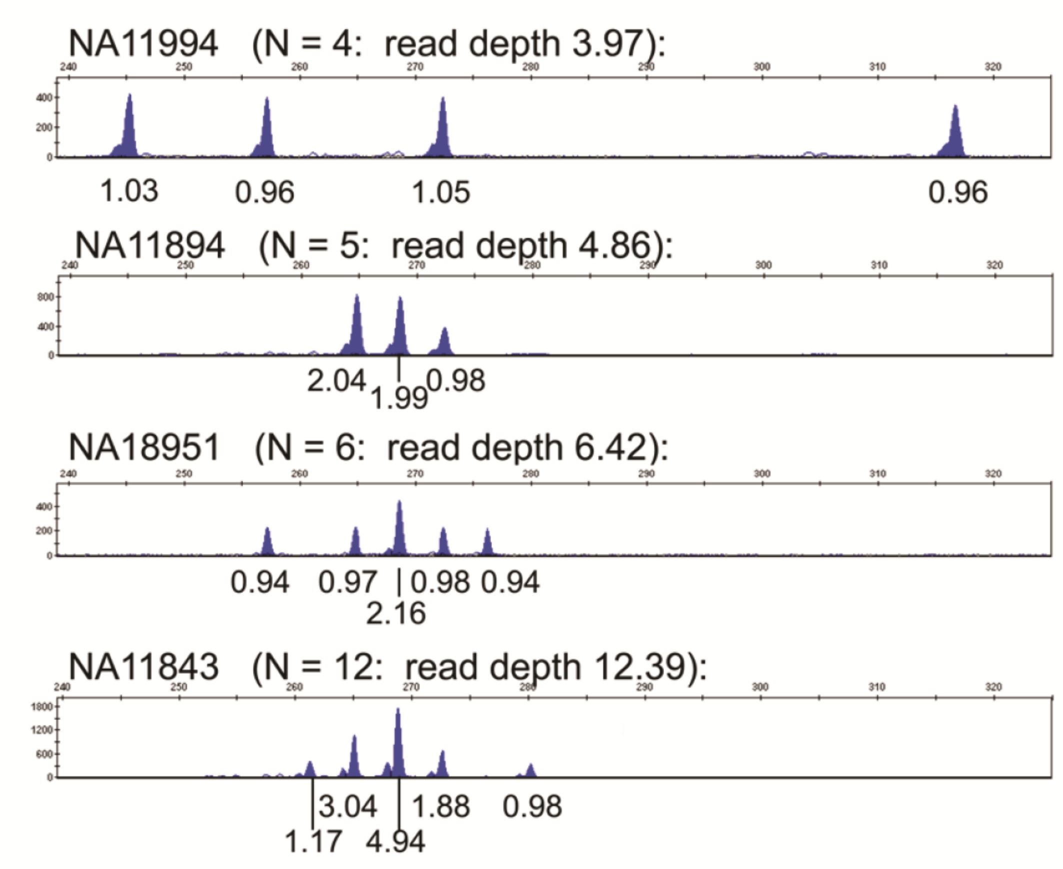

Supplementary Figure S1. Microsatellite profiling of *AMY1* copy number.

AMY1 microsatellite profiles illustrating concordance with copy number measured using PRT and read-depth analyses. A value for the relative area is shown under each peak, assuming that the total peak area is split according to the integer *AMY1* copy number indicated. Although the relative areas conform very accurately to the predictions, these values only predict *relative* ratios between *AMY1* repeats, so that (for example) without additional information from other tests such as PRT, NA11894 could have a copy number of 10, with the three peaks being derived from 4, 4 and 2 copies, rather than from 2, 2 and 1.

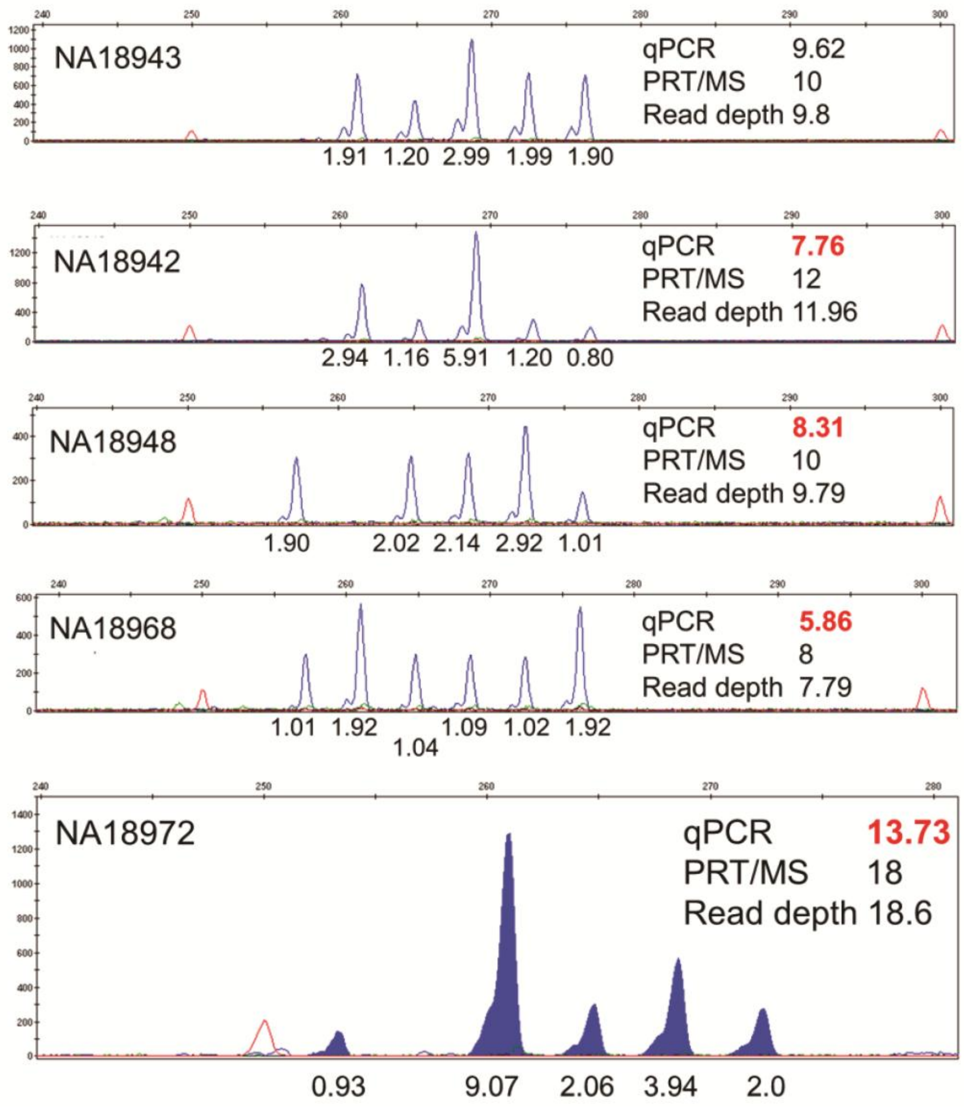

Supplementary Figure S2. ***AMY1*** **microsatellite profiles for JPT HapMap samples.**

All assays agree on a value of 10 for NA18943, and the remaining four panels show samples (NA18942, NA18948, NA18968, NA18972) in which microsatellite, read-depth and PRT data are at variance with the qPCR data of Perry *et al.* (red highlight). In all cases, the estimated *AMY1* copy number attributable to that peak is shown below each microsatellite peak, assuming that the total integer copy number favoured by read depth and PRT is split in proportion to the observed microsatellite peak areas. Red peaks are size standards at 250 and 300nt.

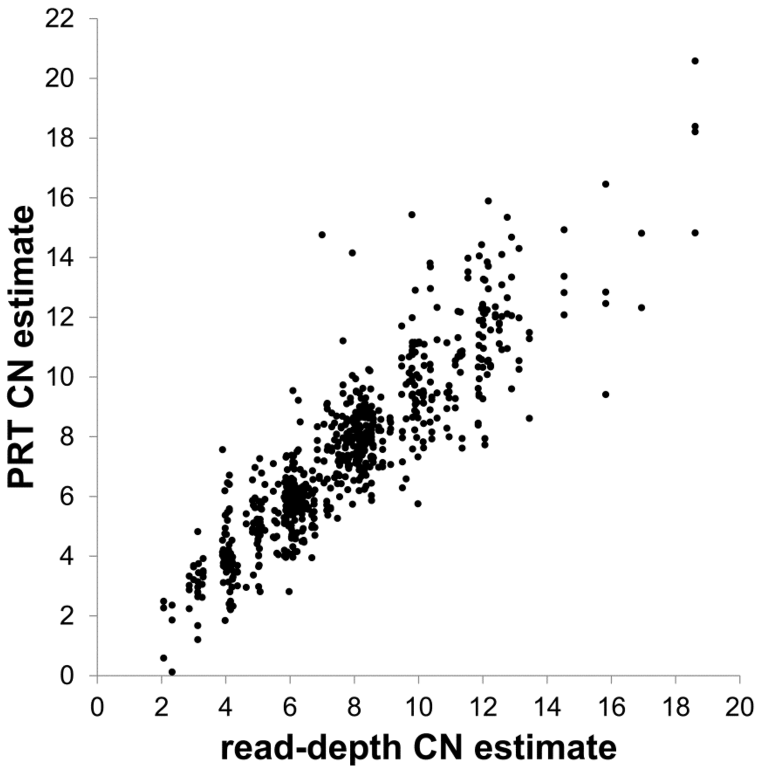

Supplementary Figure S3. Concordance of PRT and read-depth estimates of *AMY1* copy number (CN).

Results from 737 PRT_ref12-based *AMY1* CN estimates are plotted against CN estimates based on read-depth for 208 HapMap samples (r^2 = 0.845).

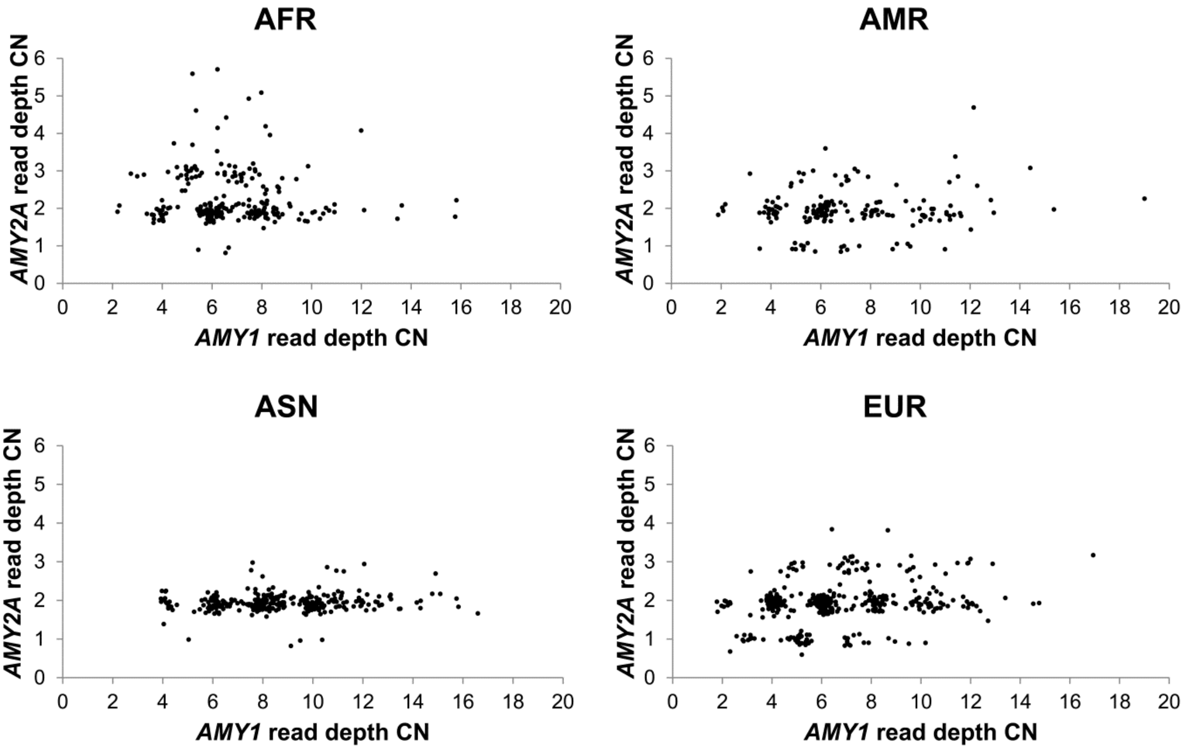

Supplementary Figure S4. Read-depth analysis of *AMY1* and *AMY2A* copy number.

Analysis of read-depth estimates of *AMY2A* versus *AMY1* copy number (see also Figure 2c) shown separately according to the major regional groups of the 1000 Genomes Project: AFR (African = ASW, LWK, YRI, N = 228), AMR (American = CLM, MXL, PUR, N = 175), ASN (Asian = CHB, CHS, JPT, N = 279) and EUR (European = CEU, FIN, GBR, IBS, TSI, N = 365).

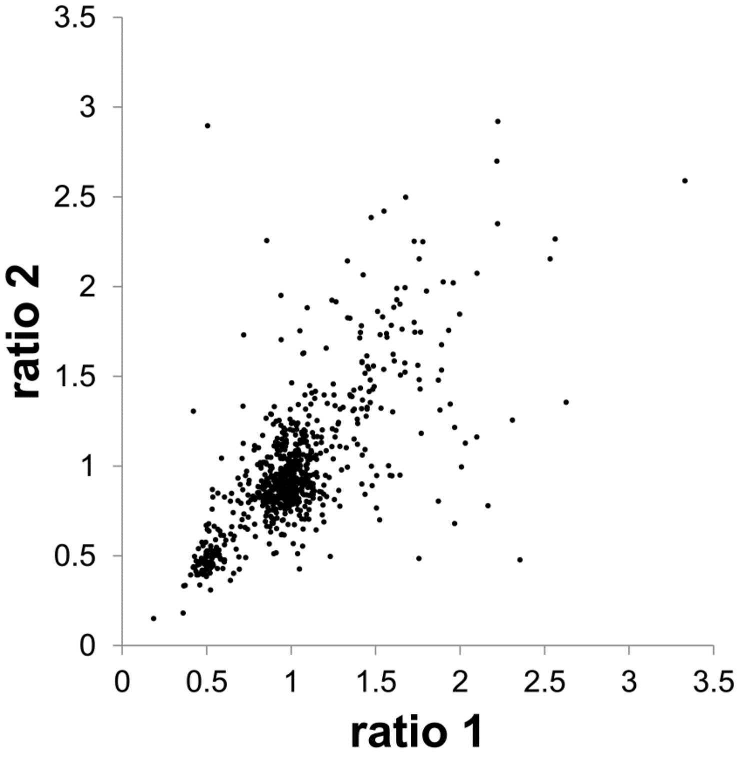

Supplementary Figure S5. Ratios of PRT_ref12 to PRT_ref1 measurements for 739 samples.

AMY1 copy number was measured using both PRT_ref1 and PRT_ref12, and the apparent copy number of *AMY1* from the two methods was compared by deriving the ratio (PRT_ref12 CN)/(PRT_ref1 CN). PRT_ref12 measures the ratio of *AMY1* CN against a chromosome 12 reference, and PRT_ref12 measures *AMY1* CN against an *AMY2A* reference locus; this secondary ratio (PRT_ref12 CN)/(PRT_ref1 CN) is therefore an indirect measurement of the representation of *AMY2A* relative to the chromosome 12 reference locus. This ratio was independently measured twice for each sample. Although most samples have ratios that cluster around a value of 1.0, indicating an *AMY2A* CN of 2, more than 10% of samples have a reproducible ratio of about 0.5, indicating an *AMY2A* CN of 1, and there is evidence that some samples have reproducibly high ratios indicating duplication of *AMY2A*.

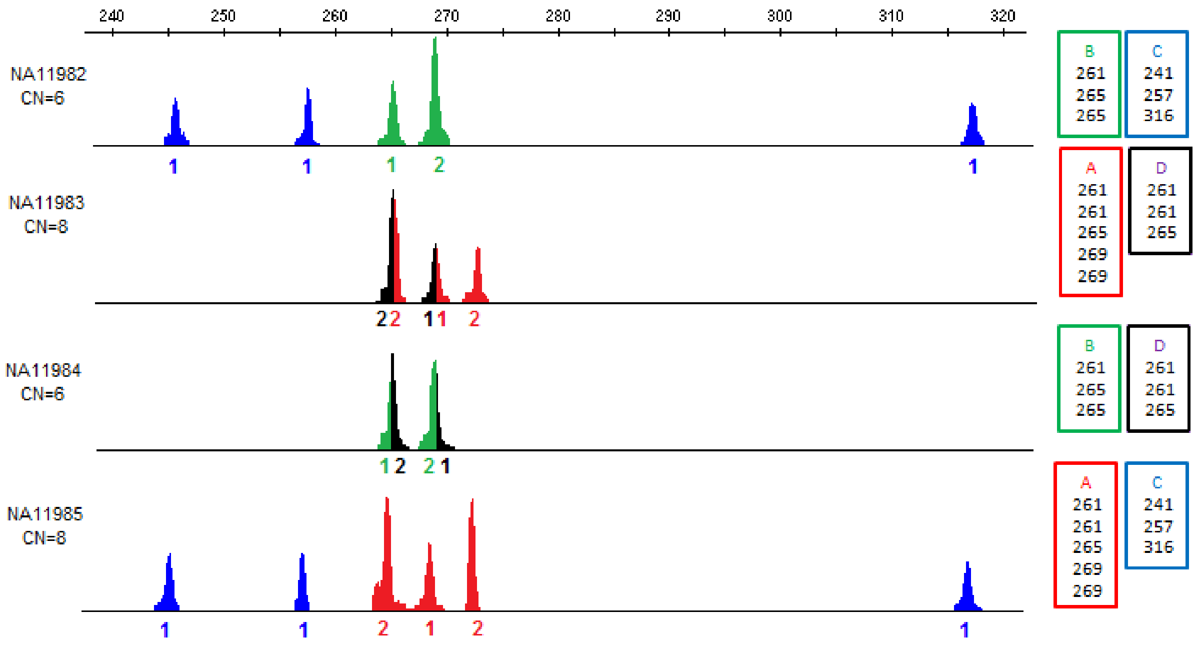

Supplementary Figure S6. Example of the use of *AMY1* microsatellite alleles to infer segregation in four sibs from CEPH pedigree 1362.

The colour coded parental haplotypes are shown as microsatellite traces with the alleles highlighted. The numbers under the peaks indicate the number of alleles inferred as present in each peak. Analysis of the parents and grandparents (not shown) confirms transmission of haplotypes A and B from his parents to the father NA10860, and haplotypes C and D from her parents to the mother NA10861. These four children (NA11982-NA11985), who between them illustrate all four possible combinations, allow the unambiguous resolution of parental haplotypes A-D, each of which carries an odd number (either 3 or 5) of copies of *AMY1*. Detailed segregation information on CEPH pedigrees can be found in Dataset S3.

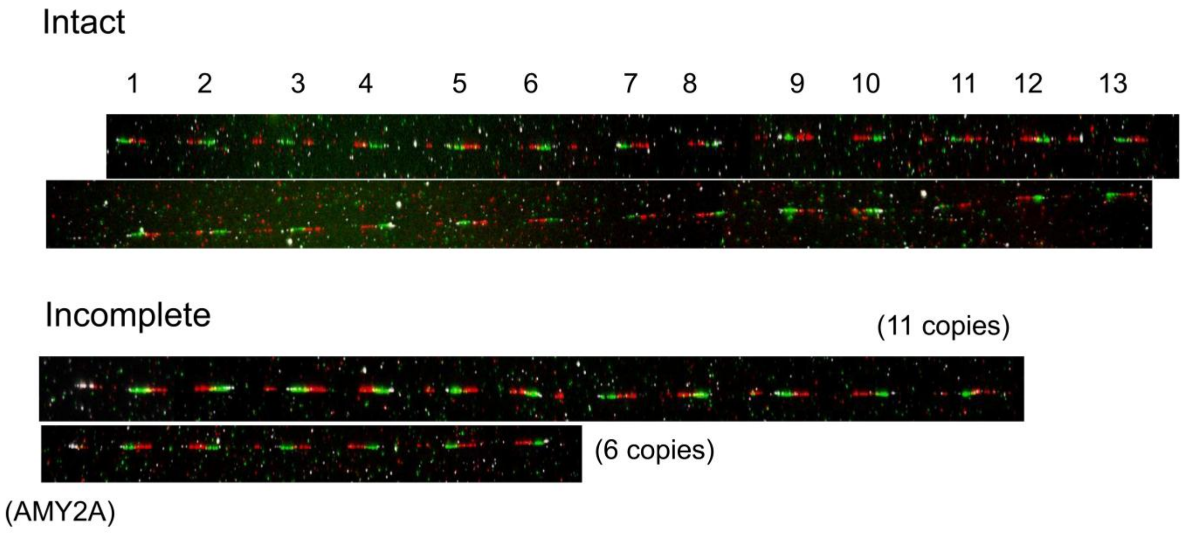

Supplementary Figure S7. Further examples of combed DNA fibre-FISH images from the 13-copy haplotype of NA18972 (see also Figure 6).

There are two ("Intact") examples of full-length structures in which all 13 repeats of *AMY1* can be seen. In the "Incomplete" structures below, the telomeric extremity of the array is marked by hybridization to a probe for *AMY2A* (white). The spacing and orientations of *AMY1* repeats observed are consistent with the corresponding repeats from full-length 13-copy haplotypes.

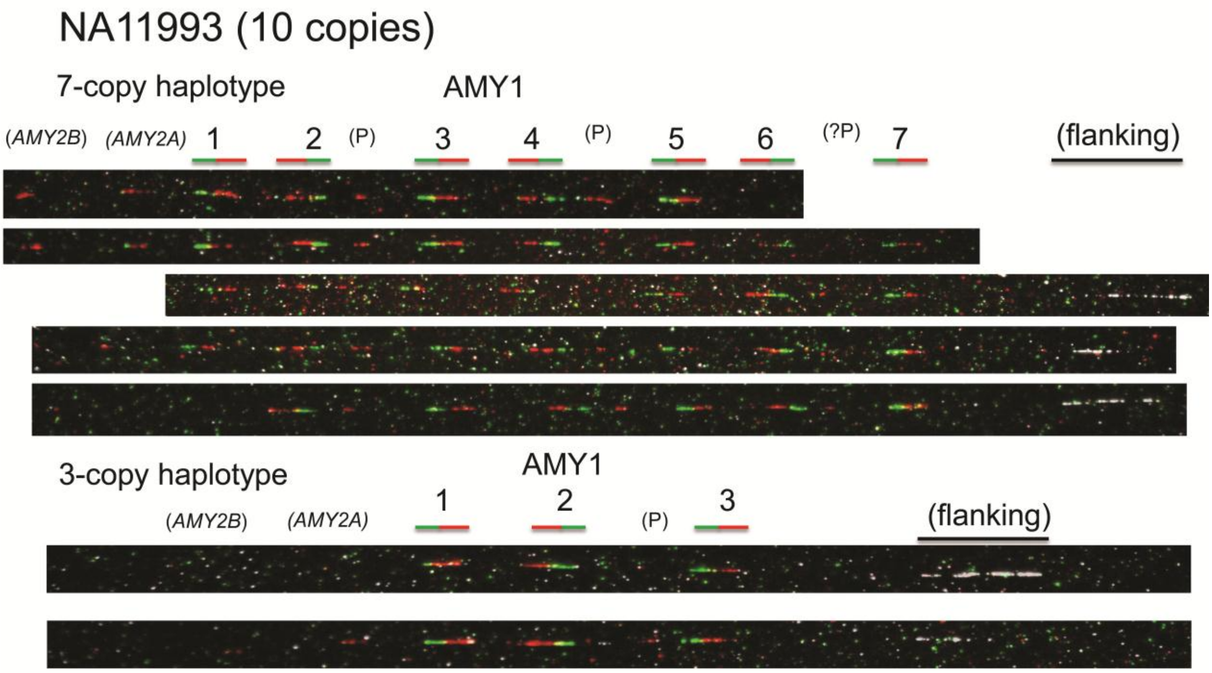

Supplementary Figure S8. Fibre-FISH on combed DNA, confirming 10 copies of *AMY1* in sample NA11993, with haplotype copy numbers of 7 and 3.

The gene probes are as used in Figure 6 and by Perry *et al.* with the upstream (ERV) probe shown in green and the *AMY1* gene probe in red. Putatively cross-hybridizing *AMY2B*, *AMY2A* and pseudogene (P) sequences are also indicated. The additional probe (white) is from a large-insert clone marking the flanking DNA on the centromeric side of the array. Two of the images of the 7-copy haplotype appear to be incomplete, presumably from broken strands; the full-length 7-copy haplotype is about 400kb in length from *AMY2B* to the last *AMY1* repeat.

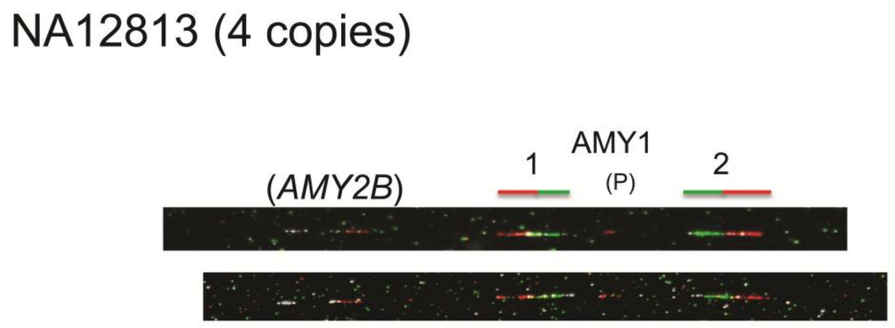

Supplementary Figure S9. Consistency of fibre-FISH analysis of NA12813 with homozygosity for 2-copy haplotypes with deletion of *AMY2A*.

In addition to the ERV probe (green) and *AMY1* gene probe (red), a probe specific for *AMY2A* was used (white). The locations of the faint signals from this probe suggest that it is cross-hybridizing to the upstream regions of *AMY2B* and *AMY1*; similarly, there is faint signal from the (red) *AMY1* gene probe at both *AMY2B* and the pseudogene (P). These putative cross-hybridizing sequences are more than 90% identical to the probes used.

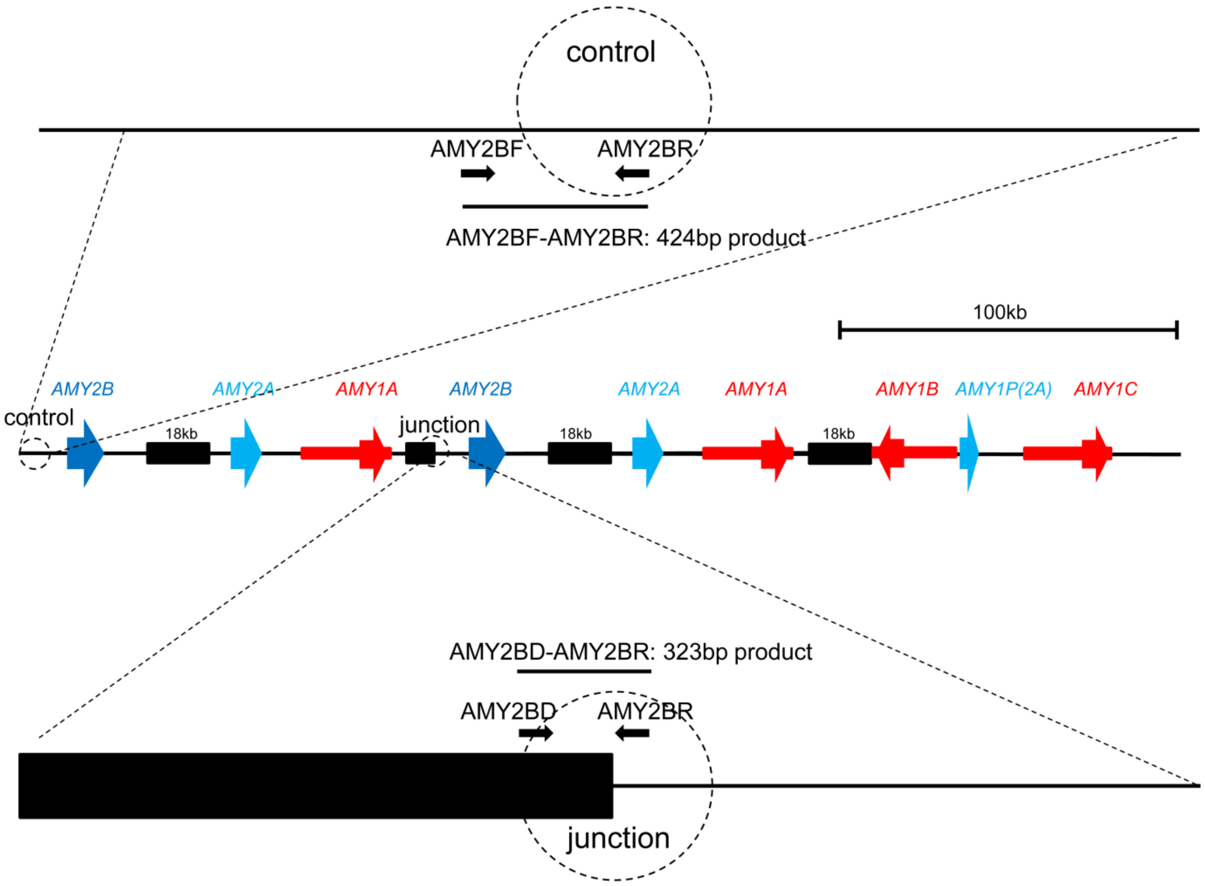

Supplementary Figure S10. Junction fragment assay for the *AMY2A/2B* duplication allele.

The central image shows a full reconstruction of the inferred structure for an *AMY2A/2B* duplication allele with 4 copies of *AMY1* (compare Figure 4c). Details show the region of the new junction, at which primers AMY2BD and AMY2BR can amplify (in carriers) a product of 323bp, and the corresponding unrearranged sequence upstream of AMY2B, at which primers AMY2BF and AMY2BR amplify a 424bp product (from all individuals).
